# Human stem cell models for Marfan syndrome: a *brief overview of the rising star in disease modelling*

**DOI:** 10.3389/fcell.2024.1498669

**Published:** 2025-01-03

**Authors:** Jeffrey Aalders, Laura Muiño Mosquera, Jolanda van Hengel

**Affiliations:** ^1^ Medical Cell Biology Research Group, Department of Human Structure and Repair, Faculty of Medicine and Health Sciences, Ghent University, Ghent, Belgium; ^2^ Center for Medical Genetics, Ghent University Hospital, Belgium and Department of Biomolecular Medicine, Faculty of Medicine and Health Sciences, Ghent University, Ghent, Belgium; ^3^ Department of Pediatrics, Division of Pediatric Cardiology, Ghent University Hospital, Ghent, Belgium

**Keywords:** human pluripotent stem cells, Marfan syndrome, disease modelling, *in vitro*, aortopathy, cardiomyopathy

## Abstract

The introduction of pluripotent stem cells into the field of disease modelling resulted in numerous opportunities to study and uncover disease mechanisms in a petri dish. This promising avenue has also been applied to model Marfan syndrome, a disease affecting multiple organ systems, including the skeletal and cardiovascular system. Marfan syndrome is caused by pathogenic variants in *FBN1*, the gene encoding for the extracellular matrix protein fibrillin-1 which ensembles into microfibrils. There is a poor genotype-phenotype correlation displayed by the diverse clinical manifestations of this disease in patients. Up to now, 52 different human pluripotent stem cells lines have been established and reported for Marfan syndrome. These stem cells have been employed to model aortopathy, skeletal abnormalities and cardiomyopathy *in vitro*. These models were able to recapitulate key features of the disease that are also observed in patients. The use of pluripotent stem cells will help to uncover disease mechanisms and to identify new therapeutic strategies in Marfan syndrome.

## Introduction

Marfan syndrome (OMIM: 154700) is a connective tissue disorder affecting multiple organ systems. Patients with Marfan syndrome display a wide range of clinical features with diverse clinical severity that could significantly add to morbidity and mortality. Skeletal features include long limbs, tall stature and arachnodactyly, scoliosis, pectus carinatum and pectus excavatum (Pyeritz, 2000). The most important cardiovascular manifestations include aortic root dilatation, which untreated can lead to aneurysm and dissection, and mitral valve prolapse (Groth et al., 2018). Other known but less prevalent cardiovascular problems are pulmonary artery dilatation, ventricular and atrial arrythmias and cardiomyopathy (Demolder et al., 2020; Isekame et al., 2016; Lazea et al., 2021). Besides the skeletal- and cardiovascular manifestations, the ocular system is affected with lens luxation (ectopia lentis) and myopia being the most important ophthalmological features (Kinori et al., 2017; Sharma et al., 2002). There is a great variability in the clinical expression of Marfan syndrome, ranging from mild to severely affected patients. Even within the members of the same family, carrying the same pathogenic variant, a wide range of clinical presentations have been described (Aalberts et al., 2010; Ergoren et al., 2019; Li et al., 2016). Both morbidity and mortality related to Marfan syndrome progresses with age.

Marfan syndrome is caused by pathogenic variants in the fibrillin 1 gene (*FBN1)*. This gene is positioned at chromosome 15q21.1. Fibrillin-1 is a large glycoprotein of 350 kDa which assembles into microfibrils that are important in elastic tissues. Microfibrils are important for the structural integrity of the connective tissue and provide tensile strength (Sakai et al., 1986). Deposition of elastin on microfibrils occurs during development and thus explains the close association between these two proteins (Milewicz et al., 2021). Furthermore, microfibrils provide an important substrate for cell adhesion through interaction with integrins, more specifically integrins α5β1, integrin αVβ3 and integrin αVβ6 (Bax et al., 2003; Jovanovic et al., 2007). Besides structural mechanical support, microfibrils are involved in the control of cell signalling pathways and play an important role in bioavailability of growth factors by binding latent transforming growth factor beta (TGFβ) binding proteins and bone morphogenetic protein (BMP) (Sengle and Sakai, 2015).

To date more than 3,305 different variants have been described in *FBN1* gene, in all 65 coding exons (ClinVar: *FBN1*, Landrum et al. (2014)). Approximately 25% of all the *FBN1* pathogenic variants are *de novo* (Chiu et al., 2014). There are different types of pathogenic variants, of which missense variants are the most abundant. Most commonly these missense variants disrupt one of the calcium binding EGF-domains by substituting or inserting a cysteine residue or disrupting one of the amino acids of the calcium binding sequence ([D]-X-[D/N]-[E/H]-Xm-[D/N]-Xn-[Y/F]). These changes influence either the disulfide-bridge formation or calcium binding which are essential for correct fibrillin-1 domain conformation (Arnaud et al., 2021). Besides affecting the function, these modifications also leave the protein more vulnerable for proteolysis. Other types of pathogenic variants included nonsense, splice-site variants, and small in-frame or, frameshift insertions and deletions (Caspar et al., 2018; Stenson et al., 2003). Most of the described pathogenic variants in *FBN1* are heterozygous, homozygous is very rare and are associated with a severe phenotype (Arnaud et al., 2017). Fibrillin-1 haploinsufficiency causes Marfan syndrome in approximately 10%–15% of patients (Arnaud et al., 2021). Some studies suggest that haploinsufficient variants such as nonsense or frameshift and variants deleting a cysteine residue are associated with more severe clinical features and involvement of the aorta (Arnaud et al., 2021; Takeda et al., 2018).

Genotype-phenotype associations are however challenging because of the large variation in clinical expression. The most consistent genotype-phenotype correlation has been the association of severe and often early-onset of Marfan syndrome with pathogenic variants in exon 25 to 33 (Arnaud et al., 2021; Stheneur et al., 2011). Sex effects impact the clinical manifestations in Marfan syndrome. Males are observed to have a more severe cardiovascular presentation, including increased incidence of aortic dissections irrespective of the type of pathogenic variant (Arnaud et al., 2021; Détaint et al., 2010). Skeletal features were more frequently observed in female (Arnaud et al., 2021). Ectopia lentis and mitral valve prolapse presented a similar incidence in both sexes (Arnaud et al., 2021; Détaint et al., 2010; Du et al., 2021).

## Patient-specific stem cell models as a platform for personalized medicine

Besides clinical research, Marfan syndrome mouse models have been undoubtedly the most important tool for advancing our knowledge of the underlying pathophysiology in Marfan syndrome so far, although other species, such as zebrafish are increasingly gaining importance (Yin et al., 2021). Advancements in human pluripotent stem cells (hPSC) (Takahashi et al., 2007) could provide important human cell models for modelling the various clinical features of Marfan syndrome. Together with important knowledge gained from animal studies, hPSCs models could be applied, for instance in preclinical trials. Moreover, differences between animal models and humans, due to genetic- and physiologic differences form an important barrier for translating findings from the lab into the clinic. hPSCs have the potential to address these issues. The cellular models for Marfan syndrome can help to uncover disease mechanisms and aid in the drug discovery. Especially since hPSCs can phenocopy the human response to novel drugs, which might be different than the responses to drug observed in animals, highlighting the translational value of hPSCs. Also drug responses and toxicity could be evaluated in patient specific manner, allowing to identify important differences between individuals that can be translated into a more personalized clinical management. These insights could for instance be used as a tool for risk stratification and decision making in clinical management.

The hPSC technology provides a new tool for personalized medicine in Marfan syndrome. Using this technology, patient’s DNA with specific pathogenic variants in *FBN1* could be studied on an individual level. hPSCs can be generated from a single donation of somatic cells by patients in the form of a blood-or urine sample or a tissue biopsy and can be differentiated to all the cells of the three germ layers, providing an unlimited resource to study. Numerous directed differentiation protocols have been established and optimized, including those to cardiomyocytes and vascular smooth muscle cells (Burridge et al., 2014; Lian et al., 2013). These patient-specific models can be interrogated extensively and compared to isogenic controls and hPSC models from other patients with Marfan syndrome carrying a different pathogenic variant. The contribution of the abnormal fibrillin-1 protein, as well as other elements that can contribute to the disease severity can be studied using these *in vitro* models.

## Standardization and quality control of pluripotent stem cells

In 2006, Yamanaka and Takahashi demonstrated for the first time that mouse embryonic fibroblasts (MEFs) could be reprogrammed to mouse induced pluripotent stem cells (iPSC) (Takahashi and Yamanaka, 2006). They systematically screened 24 candidate genes, of which four factors, being Oct3/4, Sox2, c-Myc, and Klf4 were essential in the transduction process. One year later, in 2007, the group of Yamanaka was able to generate human iPSCs from human dermal fibroblasts (Takahashi et al., 2007) using the same four pluripotent factors that were previously successful in the transduction of MEFs. Pluripotency of cells is defined as the ability to give rise to all three germ layers, being endoderm, ectoderm, and mesoderm. However, iPSCs are not able to differentiate into epiblast cells, meaning that they are not able to fully recapitulate an embryo. Because of the pluripotent nature of iPSCs, cells of all lineages can be obtained, including cardiomyocytes, vascular smooth muscle cells and neurons.

Before the generation of iPSC was established, embryonic stem cells (ESC) were employed. ESCs are derived from the inner cell mass of mammalian blastocysts. The first human ESC lines were derived and described in 1998 by Thomson and co-workers (Thomson et al., 1998). Similar to iPSCs, they have the ability to grow indefinitely in a pluripotent state and give rise to all cells of the three germ layers. Several commercially available human ESCs have been established, among which are H1, H7 and H9, which are available from WiCell as cell lines WA01, WA07 and WA09 respectively.

While the introduction of hPSCs propelled the field of *in vitro* research, hPSCs also displayed experimental variability that can be difficult to control. Therefore standardization in hPSC cultures and experiments is important to increase reproducibility. This challenge also attracted the attention of the industry, which developed several solutions, including pluripotency screening with for instance the PluriTest bioinformatics assay and trilineage differentiation potential using TaqMan hPSC Scorecard, both from ThermoFisher. Previously, teratoma assays were performed to determine spontaneous differentiation to all three germ layers when transplanting PSCs into an immunocompromised mice (Aoi, 2016). The novel techniques can now replace the ethically questionable teratoma assays to determine potency of pluripotent stem cells.

Other important quality controls to consider are genetic integrity and potential contamination of the cell culture. Genetic screening of PSCs is important because *in vitro* culture, especially in long term culture may induce genetic alterations (Assou et al., 2020; Närvä et al., 2010). Different options are available for the genetic screening, amongst which G-band karyotyping is most frequently employed, others are single-nucleotide polymorphism (SNP) or copy number variation (CNV) sequencing based (Jo et al., 2020). Biological contamination of bacteria, fungi or viruses are important to monitor. It is for instance recommended to routinely screen for *mycoplasma*. Lastly, it is good practice to establish the identity of hPSCs by short tandem repeat (STR) analysis. Since this STR profile is unique for every individual it could be used to track identity throughout experiments, for instance to compare somatic cells with reprogrammed hPSCs.

## Isogenic controls to control genetic background

CRISPR/Cas technology facilities efficient gene editing of hPSCs. This advancement provides an important tool for *in vitro* modelling as isogenic controls can be generated. An isogenic control shares an identical genetic background and only differs in the presence or absence of the pathogenic variant. Other genetic elements, apart from the pathogenic variant, can influence the observed phenotype of the model and could be cancelled out using this isogenic approach. Alternatively, hPSCs generated from healthy individuals could function as a control. Here it could be considered to use an unaffected family member of the patient to have limited differences in genetic background. CRISPR/Cas technology could also be used to introduce different pathogenic variants in a healthy hPSC line, which allows to generate numerous hPSC lines for Marfan syndrome. This approach was used by Borsoi and co-workers to generate several hPSC lines (Borsoi et al., 2021a). The advantage of introducing a pathogenic variant in healthy donor hPSC line is that it does not require somatic cells of Marfan syndrome patients, and allows to model the effect of rare pathogenic variants, independently of the patient’s genetic background, more specifically. However, the absence of the patient’s specific genetic background might be actually necessary for full disease manifestation and the use of healthy donor hPSC line might fail to accurately phenocopy the pathogenic variant *in vitro*.

## Established pluripotent stem cells for Marfan syndrome

A comprehensive overview of the current established hPSC lines for Marfan syndrome is provided together with a brief description of their main findings and implications. The list provided here only includes stem cell lines that are registered in an online registry (either NIH stem cell registry or on the hPSC registry (hpscreg.eu) or published in peer-reviewed articles (Table 1). It is likely that more Marfan hPSC lines are already established but not yet shared with the scientific community.

**TABLE 1:** A summary of all established human pluripotent stem cell lines for Marfan syndrome that are described in peer-reviewed articles, or deposited in online stem cell registries, being NIH stem cell registry and hPSC registry. For each line, the pathogenic variant and patient information is summarized, if available. Also, original cell source, cell types obtained by directed differentiations for disease model, method of reprogramming, availability of isogenic (ISO) control, the generator of the line and the year of publication or deposition. Abbreviations: NA (not available), United States (United States of America).

Cell line name (and alternative names)	Pathogenic variant in *FBN1*	Patient information	Origin cell type	Differentiated cell types for disease model	Transduction method	ISO control	Institute (country)	Year	Ref
RGIe062-A or SI-154	Heterozygous variant of c.7712G > A (p.Cys2571Tyr)	Sex: Male	Blastocyst	Not described	NA, human embryonic stem cell	No	Reproductive Genetics Institute Chicago (United States)	2005	Verlinsky et al. (2005)
NIHhESC-10-0052 or MFS5	Not specified	Not specified	Blastocyst	Not described	NA, human embryonic stem cell	No	Stanford University (United States)	2010	NIH stem cell registry
VUBe008-A or VUB08_MFS	Heterozygous variant of c.266G>T (p.Cys89Phe) and trisomy 22	Sex: Female	Blastocyst	Not described	NA, human embryonic stem cell	No	Vrije Universiteit Brussel (Belgium)	2010	Mateizel et al. (2010)
MFSiPS cells (proband FB1121)	Intronic splice-site variant c.3839–1 G > T causes skipping of exon 31	Severe neonatal phenotype	Fibroblast	Osteogenic and chondrogenic fates	pMX retroviral vectors *SOX2*, *OCT4*, *KLF4*, and c-*MYC (Addgene)*	No	Stanford University (United States)	2012	Quarto et al. (2012a)
MFSiPS cell line (proband FB1592)	Frameshift variant c.1642del3ins20bp	Severe reduction in *FBN1* expression	Fibroblast	Osteogenic and chondrogenic fates	pMX retroviral vectors *SOX2*, *OCT4*, *KLF4*, and c-*MYC (Addgene)*	No	Stanford University (United States)	2012	Quarto et al. (2012a)
MFS5 or NIHhESC-10-0052	c.1747delC in the 5′ region (frameshift variant)	Not provided	Blastocyst	Osteogenic and chondrogenic fates	NA, human embryonic stem cell	No	Stanford University (United States)	2012	Quarto et al. (2012a)
UM89-1 PGD or NIHhESC-14-0276	Not specified	Not specified	Blastocyst	Not described	NA, human embryonic stem cell	No	University of Michigan (United States)	2014	NIH stem cell registry
*ch*HES-419	Heterozygous deletion variant, c.3536delA frameshift variant	Not provided	Blastocyst	Not described	NA, human embryonic stem cell	No	Central South University (China)	2015	Yang et al. (2015)
UM89-4 PGD or NIHhESC-16-0359	Not specified	Not specified	Blastocyst	Not described	NA, human embryonic stem cell	No	University of Michigan (United States)	2016	NIH stem cell registry
ISMMSi002-B or 60#3-1	Heterozygous variant of c.4082G > A (p.Cys1361Tyr)	Age: 28 Sex: Female. Dolichostenomelia, high narrow palate, dental crowding, hypermobile small joints, arachnodactyly, myopia, bilateral ectopia lentis, ascending aortic aneurysm, aortic dissection, aortic root replacement at age 28 years, mitral regurgitation	Fibroblast	Not described	CytoTune-iPS 2.0 Sendai Reprogramming Kit (Thermo Fisher)	No	Icahn School of Medicine at Mount Sinai (United States)	2017	Klein et al. (2017)
ISMMSi001-A or SAMEA104275576 or MFS44-E	Heterozygous variant of c.3976T > C (p.Cys1326Arg)	Sex: Female. Dolichostenomelia, high narrow palate, dental crowding, hypermobile small joints, arachnodactyly, myopia, bilateral ectopia lentis, ascending aortic aneurysm, aortic dissection, aortic root replacement at age 28; mitral regurgitation; striae	Fibroblast	Not described	Vector free reprogramming with SOX2, POU5F1, KLF4, MYC and LIN28 mRNA	No	Icahn School of Medicine at Mount Sinai (United States)	2017	hPSC registry
ISMMSi001-B or SAMEA104275577 or MFS44-16	Heterozygous variant of c.3976T > C (p.Cys1326Arg)	Sex: Female. Dolichostenomelia, high narrow palate, dental crowding, hypermobile small joints, arachnodactyly, myopia, bilateral ectopia lentis, ascending aortic aneurysm, aortic dissection, aortic root replacement at age 28; mitral regurgitation; striae	Fibroblast	Not described	Vector free reprogramming with SOX2, POU5F1, KLF4, MYC and LIN28 mRNA	No	Icahn School of Medicine at Mount Sinai (United States)	2017	hPSC registry
ISMMSi002-A or SAMEA104275578 or MFS60-12	Heterozygous variant of c.4082G > A (p.Cys1361Tyr)	Age: 28 Sex: Female. Dolichostenomelia, high narrow palate, dental crowding, hypermobile small joints, arachnodactyly, myopia, bilateral ectopia lentis, ascending aortic aneurysm, aortic dissection, aortic root replacement at age 28 years, mitral regurgitation	Fibroblast	Not described	CytoTune-iPS 2.0 Sendai Reprogramming Kit (Thermo Fisher)	No	Icahn School of Medicine at Mount Sinai (United States)	2017	hPSC registry
MFG880	Heterozygous variant of c.2638G > A (p.Gly880Ser)	Age: 31 Sex: Female. Pectus carinatum, dilated aortic root, abdominal aortic aneurysm, ectopia lentis, arachnodactyly and apical emphysema	Fibroblast	Smooth muscle cells	Retrovirus OSKM	Yes, with CRISPR/Cas9	University of Cambridge (United Kingdom)	2017	Granata et al. (2017)
MFC1242 or GM21943	c.3725G > A (p.Cys1242Tyr)	Age: 8 Sex: Male. Ascending aortic aneurysm, severe pectus excavatum, scoliosis, high narrow palate, hypermobile joints, arachnodactyly, myopia, ectopia lentis and mitral valve prolapse	Fibroblast	Smooth muscle cells	Retrovirus OSKM	Yes, with CRISPR/Cas9	University of Cambridge (United Kingdom)	2017	Granata et al. (2017)
#GM21974	Heterozygous variant of c.2581C > T (p.Arg861Ter) resulting in a premature stop codon	Age: 58 Sex: Female. Aortic dissection, myopia, ascending aortic aneurysms, pectus carinatum, high-arched plate, and dental crowding	Fibroblast	Mesenchymal stem cells, osteogenic and chondrogenic fate and smooth muscle cells	STEMCCA lentivirus	Yes, with TALENs (Addgene)	University of Macau (China)	2017	Park et al. (2017)
ENVY-C1	Knockout for *FBN1* resulting in premature termination of translation	NA	EVNY hESCs (WT)	Mesenchymal stem cells, osteogenic and chondrogenic fate and smooth muscle cells	NA, human embryonic stem cell	sgRNA expression into the BbsI sites of pSpCas9n (BB)-2A-Puro (Addgene plasmid ID: 48,141)	University of Macau (China)	2017	Park et al. (2017)
CMUi001-A or FBN1-E2130K-iPSC	Heterozygous variant of c.6388G > A (p.Glu2130Lys)	Age: 25 Sex: Male Ethnicity: Han. Aortic root aneurysm and aortic valve regurgitation	Fibroblast	Not described	Sendai virus with Oct4, Sox2, cMyc, Klf4	No	Capital Medical University (China)	2019	Li et al. (2019)
NCCDFWi001-A	Heterozygous variants c.2613A > C (p.Leu871Phe) and c.684_736 + 4del (splice variant)	Age: 26 Sex: Female Ethnicity: Han. Aortic root dilatation and mitral valve prolapse	Peripheral blood mononuclear cells (PBMCs)	Not described	CytoTune™ -iPS 2.0 Sendai Reprogramming Kit	No	Fuwai Hospital (China)	2019	Ma et al. (2020)
SZ-Marfan7 PGD, NIHhESC-20-0458	Heterozygous variant c.5437C > T (p.Gln1813Ter)	Not specified	Blastocyst	Not described	NA, human embryonic stem cell	No	Shaare Zedek Medical Centre (Israel)	2020	NIH stem cell registry
MFS1	c.5726T > C (p.Ile1909Thr)	Sex: Female. Defined as “mild” case. Severe mitral valve disease and aortic root aneurysm at age 27	Peripheral blood mononuclear cells (PBMCs)	Smooth muscle cells	Sendai virus vectors, following Fusaki protocols for PBMC	No	Stanford University (United States)	2020	Iosef et al. (2020)
MFS2	c.6783-6784delGT frameshift variant with premature stop codon	Sex: Female. Defined as “severe” case. Aortic root aneurysm at age 20, subsequent type B aortic dissection	Peripheral blood mononuclear cells (PBMCs)	Smooth muscle cells	Sendai virus vectors, following Fusaki protocols for PBMC	No	Stanford University (United States)	2020	Iosef et al. (2020)
MFS3	c.493C > T (p.Arg165Ter) with predicted nonsense-mediated decay and functional haploinsufficiency	Sex: Male. Aortic root replacement at age 16, subsequent type B aortic dissection at age 27, aortic arch surgery at age 34	Peripheral blood mononuclear cells (PBMCs)	Smooth muscle cells	Sendai virus vectors, following Fusaki protocols for PBMC	No	Stanford University (United States)	2020	Iosef et al. (2020)
MFS4	Not specified	Sex: Male. Aortic root aneurysm at age 18	Peripheral blood mononuclear cells (PBMCs)	Smooth muscle cells	Sendai virus vectors, following Fusaki protocols for PBMC	No	Stanford University (United States)	2020	Iosef et al. (2020)
NCCDFWi001-A-1	Heterozygous variant of c.2613A > C (p.Leu871Phe)	Age: 27 Sex: Female Ethnicity: Asian	Peripheral blood mononuclear cells (PBMCs)	Not described	Transgene free episomal plasmids	Yes, with CRISPR/Cas9	Fuwai Hospital (China)	2021	Li et al. (2021)
QDMHi001-A or PBMC20190809-01 SeVC5	Heterozygous variant of c.6772T > C (p.Cys2258Arg)	Age: 33 Sex: Male Ethnicity: Han. Aortic root aneurysm and aortic valve regurgitation	Peripheral blood mononuclear cells (PBMCs)	Not described	CytoTune®-iPS 2.0 Sendai Reprogramming Kit	No	Qingdao Municipal Hospital (China)	2021	Wu et al. (2021)
MFS patient 2	Heterozygous gene deletion resulting in haploinsufficiency	Age: 56 Sex: Female. Dilatation of root and ascending aorta and mild mitral valve prolapse with minimal regurgitation, highly arched palate, positive wrist and thumb signs, mild scoliosis, flat feet and tall stature	Fibroblast	Embryoid bodies	Polycistronic lentiviral vector (hSTEMCCA-loxP) expressing *OCT4*, *SOX2*, *KLF4*, and *cMYC*, excisable by Cre recombinase	No	University of Rome (Italy)	2021	Spitalieri et al. (2021)
LANCEi021-A or iPS-2 or iPS-MFS1	Heterozygous missense variant c.6814T > G (p.Tyr2272 Asp)	Age: 40 Sex: Female. Marfanoid habitus, myopia, and aortic dilatation/dissection	Peripheral blood mononuclear cells (PBMCs)	Cardiomyocytes	Non-integrative episomal plasmids: pEB-C5, expressing OCT4, SOX2, KLF4, CMYC and LIN28, and pEB-Tg expressing the SV40 large T antigen	No	University of São Paulo (Brazil)	2021	Borsoi et al. (2021b)
LANCEi022-A or iPS-3 or iPS-MFS2	Heterozygous deletion of c.1851_52delTG resulting in a premature stop codon (frameshift variant)	Age: 51 Sex: Male. Marfanoid habitus, myopia, aortic dilatation/dissection and ectopia lentis	Peripheral blood mononuclear cells (PBMCs)	Cardiomyocytes	Non-integrative episomal plasmids: pEB-C5, expressing OCT4, SOX2, KLF4, CMYC and LIN28, and pEB-Tg expressing the SV40 large T antigen	No	University of São Paulo (Brazil)	2021	Borsoi et al. (2021b)
LANCEi020-A-4 or IPSC16-FBN1-HI1	Induced variant using CRISPR/Cas9 monoallelic modifications in exon 2	Age: 51 Sex: Male Ethnicity: Caucasian/African/South American	Peripheral blood mononuclear cells (PBMC)	Cardiomyocytes	Non-integrating episomal plasmids pEB-C5 and pEB-Tg (Addgene), containing reprogramming factors Oct4, Sox2, Klf4, cMyc, Lin28 and SV40-T	Yes, LANCEi020-A or iPSC16	University of São Paulo (Brazil)	2021	Borsoi et al. (2021a)
LANCEi020-A-5 or IPSC16-FBN1-HI2	Induced variant using CRISPR/Cas9 monoallelic modifications in exon 2	Age: 51 Sex: Male Ethnicity: Caucasian/African/South American	Peripheral blood mononuclear cells (PBMC)	Cardiomyocytes	Non-integrating episomal plasmids pEB-C5 and pEB-Tg (Addgene), containing reprogramming factors Oct4, Sox2, Klf4, cMyc, Lin28 and SV40-T	Yes, LANCEi020-A or iPSC16	University of São Paulo (Brazil)	2021	Borsoi et al. (2021a)
LANCEi020-A-6 or IPSC16-FBN1-HI3	Induced variant using CRISPR/Cas9 monoallelic modifications in exon 2	Age: 51 Sex: Male Ethnicity: Caucasian/African/South American	Peripheral blood mononuclear cells (PBMC)	Cardiomyocytes	Non-integrating episomal plasmids pEB-C5 and pEB-Tg (Addgene), containing reprogramming factors Oct4, Sox2, Klf4, cMyc, Lin28 and SV40-T	Yes, LANCEi020-A or iPSC16	University of São Paulo (Brazil)	2021	Borsoi et al. (2021a)
LANCEi020-A-7 or IPSC16-FBN1-DN1	Induced variant using CRISPR/Cas9 heterozygous deletion exon 31	Age: 51 Sex: Male Ethnicity: Caucasian/African/South American	Peripheral blood mononuclear cells (PBMC)	Cardiomyocytes	Non-integrating episomal plasmids pEB-C5 and pEB-Tg (Addgene), containing reprogramming factors Oct4, Sox2, Klf4, cMyc, Lin28 and SV40-T	Yes, LANCEi020-A or iPSC16	University of São Paulo (Brazil)	2021	Borsoi et al. (2021a), Borsoi et al. (2021b)
LANCEi020-A-8 or IPSC16-FBN1-DN2	Induced variant using CRISPR/Cas9 heterozygous deletion exon 31	Age: 51 Sex: Male Ethnicity: Caucasian/African/South American	Peripheral blood mononuclear cells (PBMC)	Cardiomyocytes	Non-integrating episomal plasmids pEB-C5 and pEB-Tg (Addgene), containing reprogramming factors Oct4, Sox2, Klf4, cMyc, Lin28 and SV40-T	Yes, LANCEi020-A or iPSC16	University of São Paulo (Brazil)	2021	Borsoi et al. (2021a)
LANCEi020-A-9 or IPSC16-FBN1-KO	Induced variant using CRISPR/Cas9 homozygous clone for the same variant in exon 2 of HI1 (c.3delG)	Age: 51 Sex: Male Ethnicity: Caucasian/African/South American	Peripheral blood mononuclear cells (PBMC)	Cardiomyocytes	Non-integrating episomal plasmids pEB-C5 and pEB-Tg (Addgene), containing reprogramming factors Oct4, Sox2, Klf4, cMyc, Lin28 and SV40-T	Yes, LANCEi020-A or iPSC16	University of São Paulo (Brazil)	2021	Borsoi et al. (2021a)
LANCEi020-A-10 or IPSC16-FBN1-DN/DN	Induced variant using CRISPR/Cas9 compound heterozygous for exon 31 deletion	Age: 51 Sex: Male Ethnicity: Caucasian/African/South American	Peripheral blood mononuclear cells (PBMC)	Cardiomyocytes	Non-integrating episomal plasmids pEB-C5 and pEB-Tg (Addgene), containing reprogramming factors Oct4, Sox2, Klf4, cMyc, Lin28 and SV40-T	Yes, LANCEi020-A or iPSC16	University of São Paulo (Brazil)	2021	Borsoi et al. (2021a)
ZJUi005-A or WXG-iPSC-C6	Heterozygous variant of c.6734G > A (p.Pro2245Tyr)	Age: 47 Sex: Male. Aortic valve insufficiency, aneurysm of ascending aorta and left ventricular hypertrophy	Fibroblast	Not described	Sendai virus (KOS, c-MYC, KLF4)	No	Zhejiang University (China)	2021	Pan et al. (2021)
ZZUSAHi003-A	Heterozygous variant of c.2939G > A (p.Cys980Tyr)	Age: 26 Sex: Female Ethnicity: Han	Peripheral blood mononuclear cells (PBMCs)	Not described	Sendai reprogramming kit	No	Zhengzhou University (China)	2021	Qin et al. (2021)
FUHSi001-A or GD09006	Heterozygous variant of c.1858C > T (p. Pro620Ser)	Age:38 Sex: Male Ethnicity: Han Chinese	Peripheral blood mononuclear cells (PBMCs)	Not described	CytoTune®-iPS 2.0 Sendai Reprogramming Kit	No	Fudan University (China)	2022	Lin et al. (2022)
INSRMe013-A or STR-I-301-MFS	Not specified	Sex: Female	Blastula with ICM and Trophoblast	Not described	NA, human embryonic stem cell	No	Inserm (France)	2022	hPSC registry
ICSSUi001-A	Heterozygous variant of c.7897T > G (p.Cys2633Gly)	Age:15 Sex: Male Ethnicity: Han Chinese	Peripheral blood mononuclear cells (PBMCs)	Not described	Episomal vectors containing four classical “Yamanaka” factors, LIN28 and shRNA targeting human TP53 (Addgene #27077, 27,078, 27,080)	No	Soochow University (China)	2022	Yu et al. (2022)
ZJUi009-A or ZJULLi001-A	Not specified	Sex: Male	Fibroblast	Not described	Sendai virus	No	Zhejiang University (China)	2022	hPSC registry
CMGANTi005-A or iPSC-PBMC_MFS_FBN1_MCE-KB_C8	Heterozygous variant of c.7754T > C (p.Ile2585Thr)	Age: 68 Sex: Female Ethnicity: Caucasian	Peripheral blood mononuclear cells (PBMCs)	Smooth muscle cells	CytoTune®-iPS 2.0 Sendai Reprogramming Kit	No	Antwerp University (Belgium)	2023	Van Den Heuvel et al. (2023)
CMGANTi008-A or iPSC_MFS_FBN1_Fi930129_C8	Heterozygous variant of c.5372G>A (p.Cys1791Tyr)	Age: 15 Sex: Male Ethnicity: Caucasian	Fibroblast	Smooth muscle cells	CytoTune®-iPS 2.0 Sendai Reprogramming Kit	No	Antwerp University (Belgium)	2023	Peeters et al. (2023)
UGENT001-A or UGENT-MFS003	Heterozygous variant of c.7754T > C (p.Ile2585Thr)	Age: 34 Sex: Male Ethnicity: Caucasian	Renal epithelial cells from urine	Cardiomyocytes	CytoTune®-iPS 2.0 Sendai Reprogramming Kit	Yes, with CRISPR/Cas9	Ghent University (Belgium)	2023	Aalders et al. (2023)
DE35	Heterozygous variant of c.1837+5G > C (intronic variant)	Not specified	Fibroblast	Smooth muscle cells	CytoTune®-iPS 2.0 Sendai Reprogramming Kit	No	University of Cambridge (United Kingdom)	2023	Davaapil et al. (2022)
DE37	Unknown – diagnosis was based on clinical criteria	Reduced fibrillin-1 deposition in patient fibroblasts	Fibroblast	Smooth muscle cells	CytoTune®-iPS 2.0 Sendai Reprogramming Kit	No	University of Cambridge (United Kingdom)	2023	Davaapil et al. (2022)
DE119	Heterozygous variant of c.1051C > T (p.Gln351Ter)	Not specified	Fibroblast	Smooth muscle cells	CytoTune®-iPS 2.0 Sendai Reprogramming Kit	No	University of Cambridge (United Kingdom)	2023	Davaapil et al. (2022)
FJMAi001-A	Heterozygous variant of c.2777G>A (p.Cys926Tyr)	Sex: Male Ethnicity: Han Chinese. Age: 57 Dissection of aorta, thoracic aortic aneurysm	Peripheral blood mononuclear cells (PBMCs)	Not described	Episomal non-integrating vector for SOX2, KLF4, MYC, POU5F1, LIN28A	No	Fujian Academy of Medical Sciences (China)	2024	(Liu et al., 2024)
JHUi005-A or HFD1	Heterozygous variant of c.3338–2A>C (intronic variant)	Age: 5–9 Sex: Female	Fibroblast	Not described	CytoTune®-iPS 2.0 Sendai Reprogramming Kit	No	Johns Hopkins University (United States)	2024	Hall et al. (2024)
SCVIi128-A	Heterozygous variant of c.1942C>A (p.Cys 1942X)	Age: 26 Sex: Female Ethnicity: Caucasian	Peripheral blood mononuclear cells (PBMCs)	Not described	CytoTune®-iPS 2.0 Sendai Reprogramming Kit	No	Stanford Cardiovascular Institute (United States)	2024	Vacante et al. (2024)
SCVIi129-A	Heterozygous variant of c.1954T>C (p.Cys652Arg)	Age: 37 Sex: Male Ethnicity: South Asian	Peripheral blood mononuclear cells (PBMCs)	Not described	CytoTune®-iPS 2.0 Sendai Reprogramming Kit	No	Stanford Cardiovascular Institute (United States)	2024	Vacante et al. (2024)

The increasing number of described Marfan hPSCs, especially using the reprogramming of somatic cells, provides an important source for modelling the disease in a dish. A special article type called Lab Resource was introduced by Elsevier’s journal Stem Cell Research which provides researchers the opportunity to publish newly established pluripotent stem cell lines derived from embryos or generated through somatic cell reprogramming in detail. Furthermore, the partnership between Elsevier and the hPSC registry (https://hpscreg.eu/) further boosted transparency, standardization, and availability of hPSC for disease modelling. Remarkably, Marfan hPSCs that were not registered in the hPSC registry oftentimes did not specify essential information on the line such as pathogenic variant, age, or sex (see Table 1).

**FIGURE 1:**
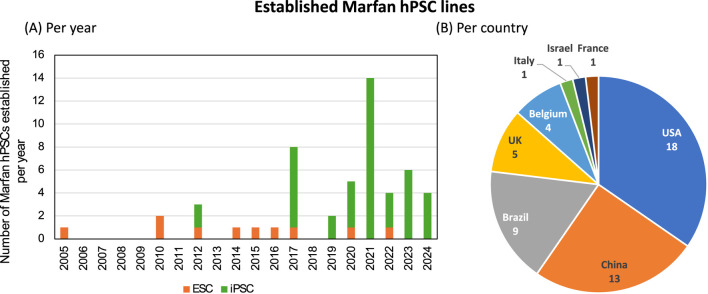
Established human pluripotent stem cell lines for Marfan syndrome. In total 52 human stem cell lines were identified in scientific literature or in online registries. **(A)** The distribution of generated stem cell lines, either human embryonic stem cells (ESC, orange) or induced pluripotent stem cells (iPSC, green) over the years. **(B)** Distribution of generated stem cell lines per country.

It is apparent that an increasing number of hPSC lines have been established for Marfan syndrome, especially the last few year (Figure 1A). Possibly due to advances in the reprogramming of somatic cells to hPSCs with robust tools such as CytoTune®-iPS 2.0 Sendai Reprogramming Kit (Thermofisher Scientific). This has facilitated researchers with the opportunity of reprogramming, without the need of viral handling and modifying viral vectors. The use of embryonic derived stem cells has been regulated more strictly over the years due to ethical concerns, which are likely be replaced completely by iPSCs in the coming decade. In total 52 different hPSC lines for Marfan syndrome were identified. The distribution of countries shows that most hPSCs were generated in The United States of America (USA), being 18, closely followed by China (13) and Brazil (9) (Figure 1B). However, this only addresses the publicly shared hPSC lines, and thus potentially not accurately reflecting the real distribution. For instance, projects with NIH funding (United States funding body) pose strict regulations and require that data, including generated hPSCs are publicly available. Whereas other funding bodies might not have the requirement to share all data publicly, potentially leading to an underrepresentation of those countries in this current overview.

## Different cellular models for Marfan syndrome

**FIGURE 2:**
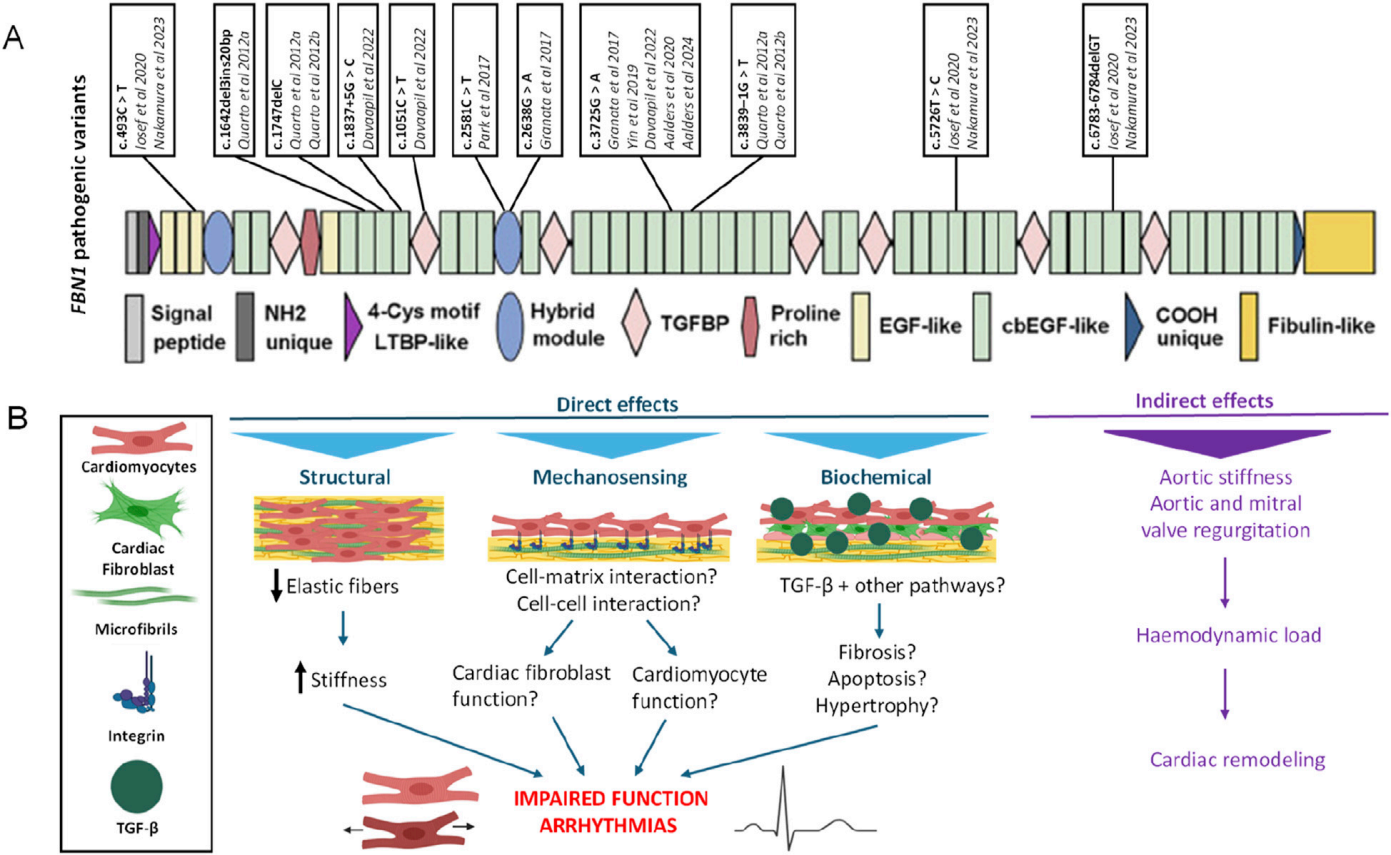
**(A)** Different pathogenic variants in *FBN1* used for the modelling of Marfan syndrome *in vitro*. **(B)** Primary functions of fibrillin-1. *FBN1* variants can have various effects which are illustrated in the context of heart function. Indirectly, by altering aortic characteristics which imposes abnormal haemodynamic loads on the heart. Or, directly, via structural, mechanosensing or biochemical pathways. For instance, by impaired elastin fibres, increased stiffness or disrupted interactions between cell and matrix or upregulation of TGFβ signalling. These direct and indirect effects on cardiomyocytes and cardiac fibroblasts could impair heart function and could induce arrhythmias. LTBP: latent TGFβ binding protein, EGF domain: epidermal growth factor-like domain, cbEGF domain: calcium binding epidermal growth factor-like domain.

Different *in vitro* models derived from hPSCs with different pathogenic *FBN1* variants have been established to study various aspects of the multi-organ affecting disease that is Marfan syndrome (Table 2; Figure 2A). The different pathogenic variants in *FBN1* used in hPSC derived *in vitro* models can have different effects, including structural, mechanosensing and biochemical effects (Figure 2B). In case of cardiac disease modelling pathogenic variants in *FBN1* can result in impaired cardiac function or arrythmia but the pathogenic variants can also impact heart function indirectly through altered hemodynamic load.

**TABLE 2:** Summary of *in vitro* cellular models for Marfan syndrome established with pluripotent stem cells to model various aspects of the disease, including aortopathy, skeletal phenotype and Marfan-related cardiomyopathy.

Modelling	Cell types used in disease model	*FBN1* variant	Main findings	Year	References
Aortopathy	Smooth muscle cells	c.2638G > A and (p.Gly880Ser) c.3725G > A (p.Cys1242Tyr)	Upregulation of MMP, increased apoptosis, reduced contractility, and dysregulated calcium handling (Figure 3B)	2017	Granata et al. (2017)
Aortopathy	Smooth muscle cells	c.2581C > T (p.Arg861Ter) and knockout for *FBN1*	Decreased contractility and impaired calcium handling. Contractility was restored by inhibiting TGFβ signalling	2017	Park et al. (2017)
Aortopathy	Smooth muscle cells	c.3725G > A (p.Cys1242Tyr)	Lack of elastin expression in *in vitro* cultures did not allow to replicate upregulation of *MFAP4* seen in human aortic tissue	2019	Yin Xiaoke et al. (2019)
Aortopathy	Smooth muscle cells	c.5726T > C (p.Ile1909Thr), c.6783-6784delGT (frameshift variant), c.493C > T (p.Arg165Ter), not specified	Downregulation of MRC2 and TAGLN, upregulation of integrin αV and MMP2 and impaired adhesion to laminin-1 and fibronectin	2020	Iosef et al. (2020)
Aortopathy	Smooth muscle cells	c.1837+5G > C (intronic variant), c.1051C > T (p.Gln351Ter), c.3725G > A (p.Cys1242Tyr), not specified	GSK3β is a promising target to decrease proteolysis and apoptosis and improved fibrillin-1 deposition (Figure 3A)	2023	Davaapil et al. (2022)
Aortopathy	Smooth muscle cells	c.5726T > C (p.Ile1909Thr), c.6783-6784delGT (frameshift variant), c.493C > T (p.Arg165Ter)	Overexpression of intregin αv resulting in activation of downstream targets (FAK/AktThr308/mTORC1). GLPG0187 shows a promising treatment that was able to reverse the levels similar to control	2023	Nakamura et al. (2023)
Skeletal	Osteogenic and chondrogenic fates	c.3839–1G > T (intronic splice-site variant), c.1642del3ins20bp (frameshift variant), c.1747delC (frameshift variant)	upregulation of TGFβ signalling in Marfan hPSCs impairs the osteogenic differentiation and could be restored by a TGFβ inhibitor	2012	Quarto et al. (2012a)
Skeletal abnormalities	Osteogenic fate	c.1747delC (frameshift variant) and c.3839-1G > T (intronic splice-site variant)	BMP2 signalling could alleviate TGFβ induced effects on osteogenesis	2012	Quarto et al. (2012b)
Skeletal abnormalities	Osteogenic and chondrogenic fates	c.2581C > T (p.Arg861Ter)and knockout for *FBN1*	Impaired and fragmented microfibrils and elastin deposition and increase SMAD2 phosphorylation	2017	Park et al. (2017)
Cardiomyopathy	Cardiomyocytes	c.3725G > A (p.Cys1242Tyr)	Lower beat-to-beat variability, impaired mechanotransduction and delayed development (Figure 2B)	2020	Aalders et al. (2020)
Cardiomyopathy	Cardiomyocytes and cardiac fibroblasts	c.3725G > A (p.Cys1242Tyr)	Decreased development of cardiomyocytes and nuclear blebbing in cardiac fibroblasts (Figure 2B)	2024	Aalders et al. (2024)

## Pluripotent stem cells used for modelling aortopathy in Marfan syndrome

In 2017, the Sinha group developed the first *in vitro* model for Marfan aortopathy (Granata et al., 2017). One of the major cell types involved in the aorta are the vascular smooth muscle cells. Depending on the location of the aorta (root, ascending, descending) the smooth muscle cells are different from another distinct mesodermal lineage. The smooth muscle cells in the descending aorta originate from paraxial mesoderm, the ascending aorta from the neural crest and the aortic root from the lateral plate mesoderm (Majesky, 2007). The different origins were reproduced by three distinct differentiation protocols used to derive smooth muscle cells from the three distinct regions of the aorta (Granata et al., 2017). The smooth muscle cells derived from the neural crest were best able to phenocopy the ascending aorta in Marfan patients and showed irregular fibrillin-1 deposition.

**FIGURE 3:**
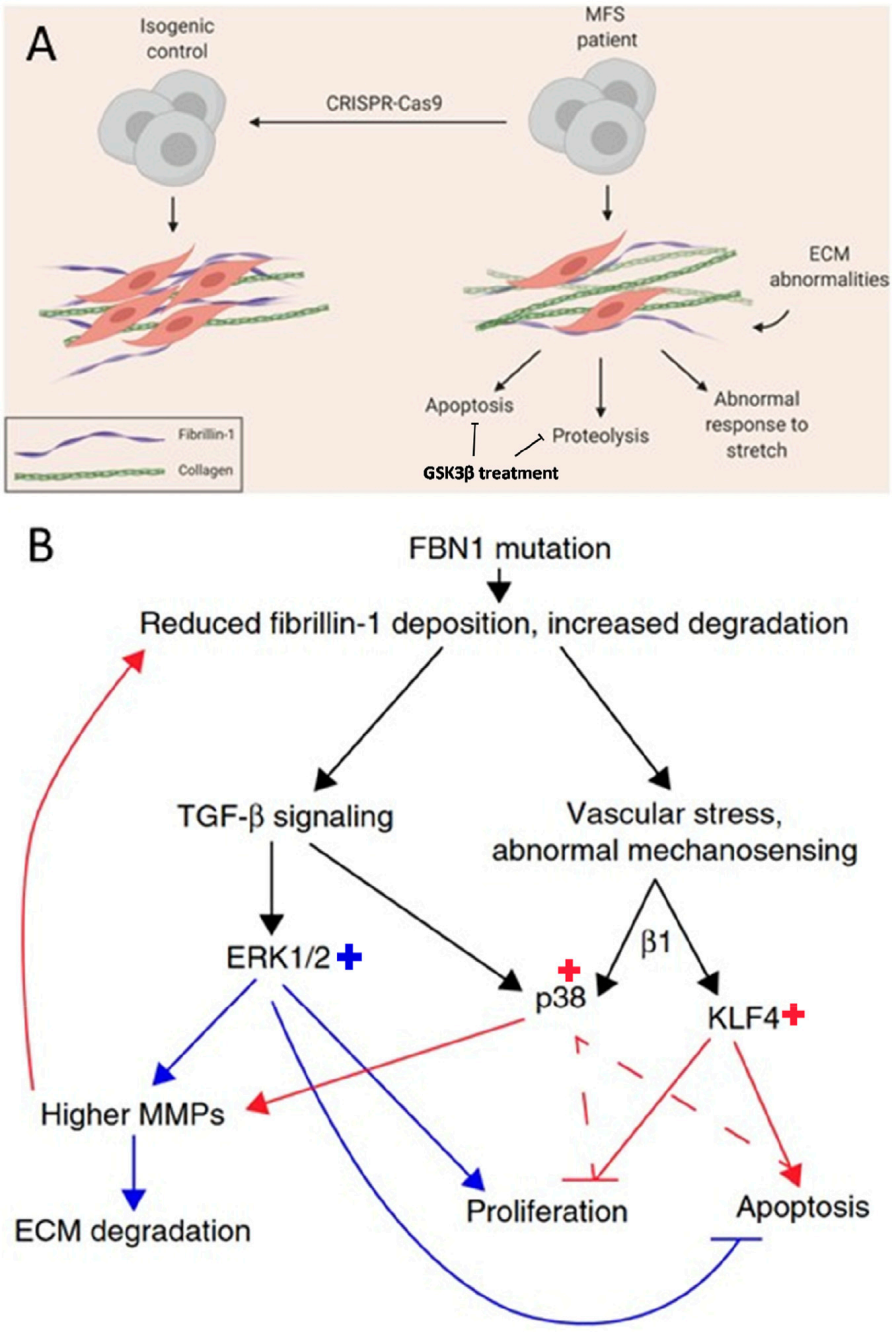
Schematic of an *in vitro* disease model for Marfan aortopathy based on pathogenic variants in *FBN1* (c.2638G > A and c.3725G > A) used in two different studies. **(A)** Comparing Marfan iPSC-derived vascular smooth muscle cells, with the isogenic control reveals abnormalities in the extracellular matrix (ECM), increased levels of apoptosis, proteolysis, and an abnormal response to stretching. GSK3β treatment could be promising to decrease proteolytic and apoptotic effects. Adapted from (Davaapil et al., 2020). **(B)** The proposed mechanism for abnormal behaviour of vascular smooth muscle cells in Marfan aortopathy through abnormal mechanosensing effects in smooth muscle cells (red arrows) and abnormal TGFβ signalling (blue arrows) resulting in increased ERK1/2, p38 and KLF4. Adapted from (Granata et al., 2017).

To evoke a phenotype in their Marfan aortopathy model the researchers made use of stretching, which could be compared to cyclic stretching of the aorta under physiological condition (Granata et al., 2017). The model revealed upregulation of TGFβ pathway, both canonical and noncanonical that results in upregulation of matrix metalloproteinase (MMP), increased apoptosis, increased matrix degradation, reduced muscle contractility and disturbed calcium handling. Three different drug treatments were evaluated in the model, being TGFβ inhibitor, losartan and doxycycline. Losartan was most effective in reducing extracellular matrix degradation and could partially rescue the diminished cell proliferation in Marfan smooth muscle cells. They also found important downstream targets, being phosphorylation of Smad, Erk, KLF4 and p38. Based on their findings they proposed disease mechanism that regulates behaviour of smooth cells in Marfan aortopathy (Figure 3). Posing cyclic stetch on the Marfan smooth muscle cells resulted in increased p38 activation, which could be resulting from integrin β1 activity, implicating a role for impaired mechanosignaling in Marfan syndrome. Employing CRISPR/Cas9 technology allowed the researchers to correct the pathogenic variant that resulted in restored TGFβ, fibrillin-1 and MMP levels. Thus, providing evidence of the role for the pathogenic variant.

Another study found upregulated *MFAP4* (microfibril-associated glycoprotein 4) gene expression upon TGFβ exposure in smooth muscle cells form aortic tissue of human and mouse (Yin Xiaoke et al., 2019). This glycoprotein MFAP4 is involved in elastic fibre assembly. These findings were also studied using a Marfan hiPSC line with a c.3725G > A *FBN1* pathogenic variant which was differentiated to neural crest derived vascular smooth muscle cells. Gene expression was evaluated for smooth muscle cells in stretched and unstretched condition. Expression of *FBN1* was higher in Marfan smooth muscle cells compared to control irrespective of the stretching condition (Yin Xiaoke et al., 2019). No differences in gene expression of *MFAP4* between hPSC derived Marfan and control smooth muscle cells could be detected and stretching did not attenuated expression of *MFAP4*. The authors observed only very little elastin expression in the *in vitro* cultures. However, some cultures that displayed detectable levels of elastin expression also showed increased *MFAP4* expression. The authors reason that increase in *MFAP4* is a disease mechanism resulting from abnormal elastin formation in Marfan syndrome, instead of a direct result of altered *FBN1* expression.

Park and co-workers established hPSC lines with a pathogenic variant in *FBN1* (c.2581C > T) and a knockout for *FBN1* for differentiation of mesenchymal stem cells to study osteogenesis and vascular smooth muscle cells to model Marfan aortopathy (Park et al., 2017). In their approach they generated an iPSC line with the variant in *FBN1* and established an isogenic control by correcting the variant using TALENs, which they termed a ‘gain-of-function’ control line. In addition, *FBN1* was knocked out in wild-type hESCs to produce a ‘loss-of-function’ line. The cellular model for Marfan syndrome revealed changes in microfibril formation, confirming the pathogenic role of *FBN1* variants. Smooth muscle cells are involved in regulating blood pressures and flow by their contractility. Marfan vascular smooth muscle cells revealed decreased sensitivity to carbachol (which increases IP3 levels and cause IP3 mediated calcium release in smooth muscle cells) as showed by decreased contractility and impaired calcium handling. Interestingly, contractility could be restored when inhibiting TGFβ signalling with SB431542. This implies that impaired contractility mainly results from dysregulated TGFβ signalling. Decreased influx of Ca^2+^ was correlated with reduced contractility observed in Marfan smooth muscle cells. Also, Marfan vascular smooth muscle cells displayed distinct gene expression compared to control lines. Smooth muscle cells derived from the Marfan hPSC line displayed a distinct transcriptome compared to the smooth muscle cells derived from the corrected hPSC line. The downregulated genes in Marfan smooth muscle cells included genes involved in cell adhesion such as collagens, the upregulated genes were associated with inflammatory responses.

In 2020, a research group from Stanford University, used hiPSCs (c.5726T > C, c.6783-6784delGT, c.493 C > T) with the aim to uncover genotype-phenotype correlations in aortopathy (Iosef et al., 2020). In their approach they used quantitative proteomics on a set of different hiPSCs derived smooth muscle cell populations, representing the range of genotypes observed in Marfan syndrome. The intended goal was to establish a model for risk stratification and clinical decision making, for example the timing of prophylactic surgery. A proteomic profile shows the final downstream product of gene transcription and provides insight into cellular function. The vascular smooth muscle cells in the aorta originate from distinct mesoderm linages, in this study smooth muscle cells from the aortic root and the ascending aorta were derived from lateral mesoderm and neural crest, respectively. The protein composition was quantified using mass spectrometry for in total four Marfan hiPSCs derived smooth muscle cells. Specific Marfan related protein expressions were discovered that also associate with lineage origin of the vascular smooth muscle cells. For instance, in Marfan MRC2 (mannose receptor C type 2) and TAGLN (transgelin) expression was reduced in lateral mesoderm derived smooth muscle cells compared to neural crest derived smooth muscle cells, while wild-type smooth muscle cells did not display lineage differences. MRC2 is a cell surface receptor that causes internalization and degradation of collagen, the authors hypothesized that it could also contribute to aberrant trafficking of extracellular matrix components. The decreased expression of MRC2 was also confirmed in Marfan patient derived smooth muscle cells, however downregulation of MRC2 was observed in both the aortic root and the ascending aorta. Decreased TAGLN expression suggest impaired contractility for Marfan smooth muscle cells derived from lateral mesoderm, recapitulating the smooth muscle cells at the location of the aortic root. The most upregulated proteins in Marfan lateral mesoderm derived smooth muscle cells were integrin αV and MMP2. In human derived aortic tissue integrin αV upregulation was also only observed in aortic root Marfan smooth muscle cells, thus phenocopied by the *in vitro* model. Altered integrin αV could point to reduced adhesion of the Marfan smooth muscle cells in the aortic root, as the upregulation might be a compensatory mechanism. Interestingly, Marfan smooth muscle cells displayed impaired adhesion to laminin-1 and fibronectin, both ligands for integrin αV, which could be caused by dysregulated cytoskeleton dynamics.

A recent publication from the Sinha group highlights the powerful potential of hPSCs in high-throughput screening and the drug discovery process (Davaapil et al., 2022). The researchers differentiated neural crest derived vascular smooth muscle cells from hiPSCs. Taking advantage of the highly proteolytic characteristics of the Marfan smooth muscle cells, one of the major features that is phenocopied in this cellular model, they established a platform for high throughput screening of compounds (Davaapil et al., 2022). The activity of MMPs, causing proteolysis, was monitored by a fluorescence-quenched gelatine substrate. Upon cleaving by MMP activity a fluorescent signal is released which can be detected by a plate reader. In total 1,022 compounds have been evaluated in a collaboration with AstraZeneca. Interestingly, GSK3β was put forward as an interesting target to alleviate the highly proteolytic effect of the Marfan smooth muscle cells, also confirmed by decreased MMP expression upon GSK3β inhibition. Other hallmarks of Marfan smooth muscle cells were increased apoptosis, decreased proliferation and impaired fibrillin-1 deposition based on the previous model of the Sinha group (Granata et al., 2017). Besides the positive effect on decreasing proteolysis, inhibition of GSK3β resulted in decreased apoptosis and improved fibrillin-1 deposition, however it did not increase proliferation of Marfan smooth muscle cells (Davaapil et al., 2022).

Another effort using hPSC derived smooth muscle cells to uncover disease mechanisms in Marfan aortopathy revealed an important role for integrin αv (Nakamura et al., 2023). Moreover, hPSC derived Marfan smooth muscle cells derived from second heart field shows overexpression of intregin αv in comparison to derivation of the neural crest of healthy hPSC derived smooth muscle cells from the second heart field. The overexpression resulted in activation of downstream targets including FAK, AktThr308 and mTORC1. The hPSC derived Marfan smooth muscle cells from the second heart field had increased proliferation rates and migration. Interestingly by using GLPG0187, an integrin antagonist, the authors were able to reverse the activity of these downstream targets in hPSC derived Marfan smooth muscle cells from the second heart field to control levels. GLPG0187 was also effective in Fbn1^C1039G/+^ mice and resulted in reduced aneurysm growth and elastin fragmentation. The findings of this study demonstrate an important role for integrin αv in the functional behaviour of smooth muscle cells and provides a potential treatment strategy in Marfan syndrome patients with GLPG0187.

## Pluripotent stem cells used for modelling skeletal abnormalities in Marfan syndrome

The Longaker group published in 2012 the first *in vitro* skeletogenic model for Marfan syndrome derived from hPSCs (Quarto et al., 2012a). Their models reveal that upregulation of TGFβ signalling in Marfan hPSCs impairs the osteogenic differentiation. This coincided with increased phosphorylated Smad2, an important downstream target of TGFβ and upregulated gene expression of *PAI-1* and *COL1A1*. The differentiation towards osteoblasts could be restored by application of a TGFβ inhibitor. Reversely, osteogenic differentiation could be blocked in wild-type hPSCs by treatment of TGFβ (10 ng/mL). Interestingly, differentiation towards chondrocytes is not affected by upregulated TGFβ signalling in Marfan. But in case of wild-type hPSCs, TGFβ treatment was necessary to efficiently differentiate to chondrocytes. Applying a TGFβ inhibitor in Marfan syndrome hPSCs could block chondrogenesis. These results combined reveals an important role for TGFβ signalling in phenotype determination. Importantly, the authors found that their models from both hESCs and hiPSC display identical phenotypes. This highlights the relevancy of hiPSCs for *in vitro* disease modelling. The authors also hypothesize that the disease mechanism of tall stature in Marfan syndrome caused by skeletal overgrowth is regulated by TGFβ signalling.

The Longaker group progressed their skeletogenic model for Marfan syndrome and discovered that BMP2 signalling could alleviate TGFβ induced effects on osteogenesis (Quarto et al., 2012b). Increased TGFβ signalling caused the cells to decrease endogenous BMP signalling. The authors revealed an increased proliferation rate for Marfan hPSCs compared to wild-type hPSCs which they attribute to increased TGFβ. Interestingly, the increased proliferation rate was normalized to wild-type hPSCs when a TGFβ inhibitor was used. Phosphorylated Smad1/5 was reduced in Marfan hPSCs compared to control, indicating declined BMP signalling. Treatment of Marfan hPSCs with TGFβ inhibitor resulted in increased phosphorylation of Smad1/5, to similar levels as wild-type but decreased Smad2 phosphorylation. Exogenous addition of BMP resulted in increased phosphorylation of Smad1/5 and allows efficient osteogenic differentiation of Marfan hPSCs. This study highlights the importance of both TGFβ and BMP signalling in the osteogenic differentiation that play important roles in the skeletal phenotype observed in patients with Marfan syndrome.

In the study of Park and co-workers with the gain- and loss-of-function cell lines also the osteogenic differentiation capacity was investigated (Park et al., 2017). Mesenchymal stem cells derived from Marfan hPSCs showed a 3-4-fold lower fibrillin-1 expression compared to the corrected ‘gain-of-function’ hPSC derived mesenchymal stem cells as established by Western blot. The difference could be explained by nonsense-mediated decay due to the pathogenic variants thus representing haploinsufficiency for fibrillin-1. This results in impaired and fragmented microfibrils and elastin deposition. Furthermore, an increase in phosphorylated SMAD2 was observed for Marfan mesenchymal stem cells which is an important downstream target of TGFβ signalling. Upregulation of TGFβ is a hallmark of Marfan syndrome which results from compromised matrix structure, TGFβ causes extracellular matrix degradation, further enhancing TGFβ signalling. Interestingly, in the TALENs corrected hPSC derived mesenchymal stem cells only limited phosphorylated SMAD2 was observed, thus repair of the pathogenic variant in *FBN1* also normalized TGFβ signalling. Upregulated TGFβ signalling results in reduced osteogenesis in Marfan mesenchymal stem cells, phenocopy the observed phenotype in patients with Marfan syndrome in this cellular model. In the corrected ‘gain-of-function’ model the researchers observed an equivalent osteogenesis potential compared to wild-type, thus a rescue effect.

## Pluripotent stem cells used for modelling Marfan-related cardiomyopathy

**FIGURE 4:**
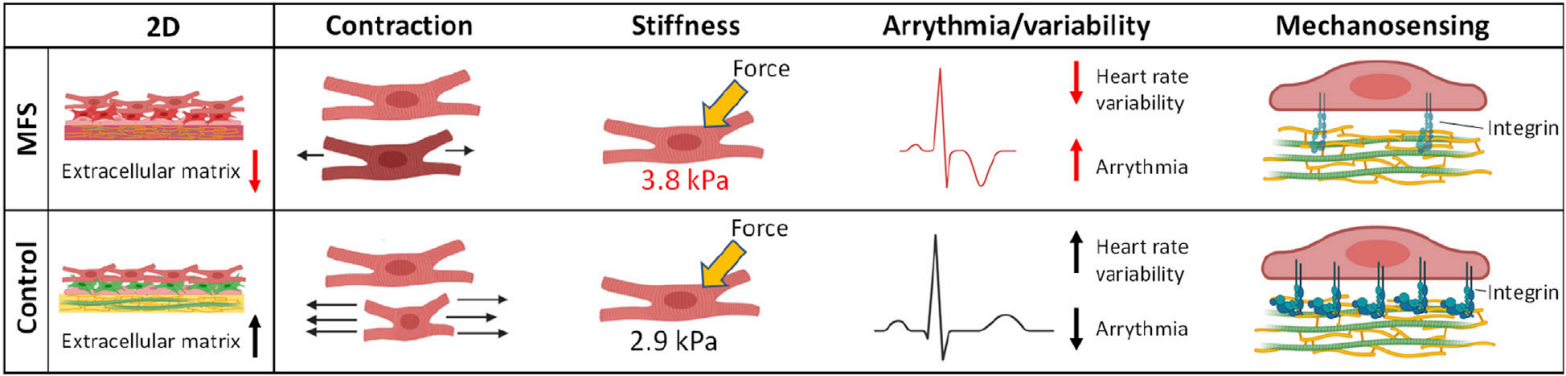
Functional abnormalities observed in the first *in vitro* model for Marfan-related cardiomyopathy using human induced pluripotent stem cell derived cardiomyocytes. These abnormalities included impaired extracellular matrix, decreased contraction amplitude, increased stiffness of cardiomyocytes and decreased heart rate variability (Aalders et al., 2020).

Intrinsic cardiomyopathy is also attributed to Marfan syndrome. In 2020, the first *in vitro* model to study the disease mechanisms for Marfan-related cardiomyopathy was established (Aalders et al., 2020) which revealed several functional abnormalities including reduced contraction, increased stiffness and decreased heart rate variability which are depicted in Figure 4. The model was generated by differentiating hiPSCs from Marfan and its isogenic control to cardiomyocytes in a 2D mono-culture. These hiPSCs have been previously used for modelling of Marfan aortopathy by the Sinha group (Granata et al., 2017).

**FIGURE 5:**
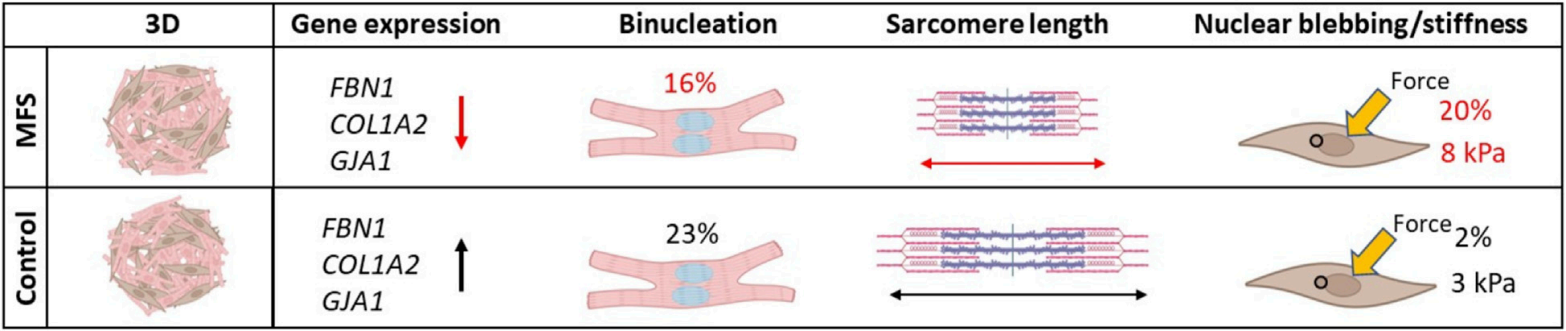
Novel disease mechanisms in Marfan-related cardiomyopathy uncovered by a 3D co-culture model with human induced pluripotent stem cell derived cardiomyocytes and cardiac fibroblasts shows a high percentage of nuclear blebbing in Marfan cardiac fibroblasts. Decreased binucleation and sarcomere length point to decreased development of cardiomyocytes as another disease mechanism at play in Marfan-related cardiomyopathy (Aalders et al., 2024).

More recently, this *in vitro* model for Marfan-related cardiomyopathy was advanced by a 3D co-culture of hiPSC derived cardiomyocytes and cardiac fibroblasts, named cardiospheres (Aalders et al., 2024). Observations made in this model may point to novel disease features that still need to be confirmed in human cardiac tissues. The Marfan cardiomyocytes showed decreased binucleation and decreased sarcomere length, which could be preserved as markers of development (Figure 5). Most intriguing was the high frequency of nuclear blebbing observed in the Marfan cardiac fibroblasts which correlated with increased stiffness of the nuclear area in these cells. Combined, these results suggest abnormal early development of cardiomyocytes but also an important role for cardiac fibroblasts in the disease mechanism of Marfan-related cardiomyopathy.

## Pluripotent stem cells to study microgravity effects on extracellular matrix structure and function

To uncover abnormalities in structural conformations in Marfan, microgravity was used to evoke a potential phenotype (Spitalieri et al., 2021). Embryoid body (EB) formation mimics early differentiation similar to that in embryonic tissue. The use of microgravity allows to maintain pluripotency in EBs. Microfibrils, composed of fibrillin-1, can relay physical cues from their environment. The aim of this study from Spitalieri en co-workers was to uncover if impaired fibrillin-1 in Marfan syndrome is involved in dysregulated mechanosignalling. The researchers showed that 7 day old wild-type EBs displayed increased stemness in response to microgravity stimulation. The Marfan hiPSC was derived from a patient with a deletion of the complete *FBN1* gene. The response was different for Marfan EBs, they showed reduced adaptation to microgravity. The authors hypothesized that the Marfan EBs have a deficient structure due to impaired fibrillin-1. Under normal gravity condition, that is 1 g, EB formation from Marfan hiPSCs showed macroscopic irregularities in shape, while wild-type EBs maintained round-shape in the first 2 days. Exposing the EBs to microgravity resulted in increased size and introduced more irregularities in the shape for both Marfan and control. Microgravity exposure resulted in significantly decreased gene expression for *αSMA, NCAM* and *AFP* that represent mesodermal, ectodermal and endodermal marker respectively compared to normal gravity for both Marfan and wild-type. These results indicate that microgravity can delay differentiation, however, to a lesser extent in Marfan EBs, as revealed by earlier upregulation of differentiation markers compared to wild-type. The authors also analysed gene expression related to the extracellular matrix. In wild-type EBs under microgravity condition E-cadherin, fibulin-1, COL12A1, COL8A2 and fibrillin-1 were upregulated at day 7 compared to normal gravity, while downregulated at day 14 and 22. Interestingly, only E-cadherin and COL12A1 were upregulated in Marfan EBs under microgravity conditions at day 7, reflecting the pathological effect of disrupted *FBN1*. Remarkably, while *FBN1* expression is significantly lower at day 7 in Marfan EBs under microgravity compared to normal gravity, a significant increase in *FBN1* expression was observed at day 22. The authors demonstrated that interrogating an *in vitro* model with microgravity could pronounce abnormalities and provides in interesting platform to study matrix diseases such as Marfan syndrome.

## Discussion

To date, only 11 hPSC models have been established, of which the majority of the models are employed for Marfan aortopathy (6 models), 3 models have been used to phenocopy skeletal features associated with Marfan syndrome, and 2 studies that describe *in vitro* models for Marfan-related cardiomyopathy (Table 2). These models provide promising insights in the disease mechanisms underlying Marfan syndrome.

The group of Sinha nicely demonstrated the use of hPSCs in screening for novel drug targets (Davaapil et al., 2022). In their study they first identified hallmark features for phenotypes observed in Marfan aortopathy using smooth muscle cells. These hallmark features in their model were increased apoptosis, decreased proliferation and enhanced proteolysis (Granata et al., 2017). In order to facilitate high-throughput screening, an easy and fast read-out method is preferred. Here they made use of the proteolytic activity of MMPs which degrades the extracellular matrix. By fluorescently labelling the provided matrix that is quenched upon degradation enables a suitable read-out.

An alternative approach for high throughput screening is to use a protein that is released in the culture medium as a biomarker. A fluorescently labelled antibody can be employed to quantify the amount of the specific biomarker and is compatible with high throughput. A potential biomarker could be elevated levels of MMPs (Granata et al., 2017), which have been described to result in extracellular matrix degradation in patients as an early marker of Marfan aortopathy. Taking advantage of a specific MMP biomarker will help to decide whether a novel compound is beneficial in the treatment of Marfan aortopathy.

The rapidly advancing field of *in vitro* disease modelling using hiPSC derived cells has opened the avenue for personalized medicine. Generating iPSCs from somatic cells to recreate individual specific cells facilitates high throughput screening platforms. Reducing costs and increasing throughput will enable future studies to use larger numbers of iPSCs, which allows to include a broader patient population. These advances allow to decipher the effects of different pathogenic variants in *FBN1* and other extracellular matrix variants that cause cardiomyopathy and uncover the disease mechanisms. This increase in fundamental knowledge will ultimately allow to discover novel drug targets that can be addressed to treat patients with Marfan syndrome.

Not all clinical features are yet modelled using hPSCs, for instance ectopia lentis which is associated with high morbidity in patients with Marfan syndrome. Modelling ectopia lentis *in vitro* will likely require more advanced models. Also, aortopathy and cardiomyopathy modelling would benefit from more complex model systems where for instance hemodynamic stress and fluid sheer stress can be imposed on the cellular model. In the last decade, the field of hiPSCs emerged and *in vitro* modelling have made tremendous advancements. There awaits an optimistic and promising feature of human stem cell models for Marfan syndrome that we now only have seen the beginning of.

## References

[B1] Aalberts J. J. Schuurman A. G. Pals G. Hamel B. J. Bosman G. Hilhorst-Hofstee Y. (2010). Recurrent and founder mutations in The Netherlands: extensive clinical variability in Marfan syndrome patients with a single novel recurrent fibrillin-1 missense mutation. Heart J. 18, 85–89. 10.1007/bf03091743 PMC2828568 20200614

[B2] Aalders J. Léger L. Demolder A. Muiño Mosquera L. Coucke P. Menten B. (2023). Generation of human induced pluripotent stem cell line UGENTi001-A from a patient with Marfan syndrome carrying a heterozygous c.7754T > C variant in FBN1 and the isogenic control UGENT001-A-1 using CRISPR/Cas9 editing. Stem Cell Res. 67, 103036. 10.1016/j.scr.2023.103036 36724552

[B3] Aalders J. Léger L. Van der Meeren L. Sinha S. Skirtach A. G. De Backer J. (2024). Three-dimensional co-culturing of stem cell-derived cardiomyocytes and cardiac fibroblasts reveals a role for both cell types in Marfan-related cardiomyopathy. Matrix Biol. 126, 14–24. 10.1016/j.matbio.2024.01.003 38224822

[B4] Aalders J. Léger L. Van der Meeren L. Van den Vreken N. Skirtach A. G. Sinha S. (2020). Effects of fibrillin mutations on the behavior of heart muscle cells in Marfan syndrome. Sci. Rep. 10, 16756. 10.1038/s41598-020-73802-w 33028885 PMC7542175

[B5] Aoi T. (2016). 10th anniversary of iPS cells: the challenges that lie ahead. J. Biochem. 160, 121–129. 10.1093/jb/mvw044 27387749

[B6] Arnaud P. Hanna N. Aubart M. Leheup B. Dupuis-Girod S. Naudion S. (2017). Homozygous and compound heterozygous mutations in the FBN1 gene: unexpected findings in molecular diagnosis of Marfan syndrome. J. Med. Genet. 54, 100–103. 10.1136/jmedgenet-2016-103996 27582083

[B7] Arnaud P. Milleron O. Hanna N. Ropers J. Ould Ouali N. Affoune A. (2021). Clinical relevance of genotype-phenotype correlations beyond vascular events in a cohort study of 1500 Marfan syndrome patients with FBN1 pathogenic variants. Genet. Med. 23, 1296–1304. 10.1038/s41436-021-01132-x 33731877 PMC8257477

[B8] Assou S. Girault N. Plinet M. Bouckenheimer J. Sansac C. Combe M. (2020). Recurrent genetic abnormalities in human pluripotent stem cells: definition and routine detection in culture supernatant by targeted droplet digital PCR. Stem Cell Rep. 14, 1–8. 10.1016/j.stemcr.2019.12.004 PMC6962701 31902703

[B9] Bax D. V. Bernard S. E. Lomas A. Morgan A. Humphries J. Shuttleworth C. A. (2003). Cell adhesion to fibrillin-1 molecules and microfibrils is mediated by alpha 5 beta 1 and alpha v beta 3 integrins. J. Biol. Chem. 278, 34605–34616. 10.1074/jbc.M303159200 12807887

[B10] Borsoi J. Farinha-Arcieri L. E. Morato-Marques M. Delgado Sarafian R. Pinheiro M. Veiga Pereira L. (2021a). Generation of genetically modified human induced pluripotent stem cell lines harboring haploin sufficient or dominant negative variants in the FBN1 gene. Stem Cell Res. 54, 102434. 10.1016/j.scr.2021.102434 34174776

[B11] Borsoi J. Morato-Marques M. de Araújo Tofoli F. Assis Pereira L. Farinha-Arcieri L. E. Delgado Sarafian R. (2021b). Generation of two human induced pluripotent stem cell (hiPSC) lines derived from unrelated Marfan Syndrome patients. Stem Cell Res. 54, 102407. 10.1016/j.scr.2021.102407 34062330

[B12] Burridge P. W. Matsa E. Shukla P. Lin Z. C. Churko J. M. Ebert A. D. (2014). Chemically defined generation of human cardiomyocytes. Nat. Methods 11, 855–860. 10.1038/nmeth.2999 24930130 PMC4169698

[B13] Caspar S. M. Dubacher N. Kopps A. M. Meienberg J. Henggeler C. Matyas G. (2018). Clinical sequencing: from raw data to diagnosis with lifetime value. Clin. Genet. 93, 508–519. 10.1111/cge.13190 29206278

[B14] Chiu H. H. Wu M. H. Chen H. C. Kao F. Y. Huang S. K. (2014). Epidemiological profile of Marfan syndrome in a general population: a national database study. Mayo Clin. Proc. 89, 34–42. 10.1016/j.mayocp.2013.08.022 24388020

[B15] Davaapil H. McNamara M. Granata A. Macrae R. G. C. Hirano M. Fitzek M. (2022). A phenotypic screen of Marfan syndrome iPSC-derived vascular smooth muscle cells uncovers GSK3β as a new target. Stem Cell Rep. 18, 555–569. 10.1016/j.stemcr.2022.12.014 PMC9968988 36669494

[B16] Davaapil H. Shetty D. K. Sinha S. (2020). Aortic “Disease-in-a-Dish”: mechanistic insights and drug development using iPSC-based disease modeling. Front. Cell Dev. Biol. 8, 550504. 10.3389/fcell.2020.550504 33195187 PMC7655792

[B17] Demolder A. von Kodolitsch Y. Muiño-Mosquera L. De Backer J. (2020). Myocardial function, heart failure and arrhythmia in marfan syndrome: a systematic literature review. Diagnostics 10, 751. 10.3390/diagnostics10100751 32992882 PMC7599866

[B18] Détaint D. Faivre L. Collod-Beroud G. Child A. H. Loeys B. L. Binquet C. (2010). Cardiovascular manifestations in men and women carrying a FBN1 mutation. Eur. Heart J. 31, 2223–2229. 10.1093/eurheartj/ehq258 20709720

[B19] Du Q. Zhang D. Zhuang Y. Xia Q. Wen T. Jia H. (2021). The molecular genetics of marfan syndrome. Int. J. Med. Sci. 18, 2752–2766. 10.7150/ijms.60685 34220303 PMC8241768

[B20] Ergoren M. C. Turkgenc B. Teralı K. Rodoplu O. Verstraeten A. Van Laer L. (2019). Identification and characterization of a novel FBN1 gene variant in an extended family with variable clinical phenotype of Marfan syndrome. Connect. Tissue Res. 60, 146–154. 10.1080/03008207.2018.1472589 29732924

[B21] Granata A. Serrano F. Bernard W. G. McNamara M. Low L. Sastry P. (2017). An iPSC-derived vascular model of Marfan syndrome identifies key mediators of smooth muscle cell death. Nat. Genet. 49, 97–109. 10.1038/ng.3723 27893734

[B22] Groth K. A. Stochholm K. Hove H. Andersen N. H. Gravholt C. H. (2018). Causes of mortality in the marfan syndrome (from a nationwide register study). Am. J. Cardiol. 122, 1231–1235. 10.1016/j.amjcard.2018.06.034 30149886

[B23] Hall F. D. 3rd Miller C. N. Gerecht S. Boheler K. R. (2024). Generation of an induced pluripotent stem cell line, JHUi005-A, from a Marfan Syndrome patient harboring a pathogenic c.3338-2A > C intronic splicing variant. Stem Cell Res. 79, 103475. 10.1016/j.scr.2024.103475 38941881

[B24] Iosef C. Pedroza A. J. Cui J. Z. Dalal A. R. Arakawa M. Tashima Y. (2020). Quantitative proteomics reveal lineage-specific protein profiles in iPSC-derived Marfan syndrome smooth muscle cells. Sci. Rep. 10, 20392. 10.1038/s41598-020-77274-w 33230159 PMC7683538

[B25] Isekame Y. Gati S. Aragon-Martin J. A. Bastiaenen R. Kondapally Seshasai S. R. Child A. (2016). Cardiovascular management of adults with marfan syndrome. Eur. Cardiol. 11, 102–110. 10.15420/ecr/2016:19:2 30310455 PMC6159454

[B26] Jo H. Y. Han H. W. Jung I. Ju J. H. Park S. J. Moon S. (2020). Development of genetic quality tests for good manufacturing practice-compliant induced pluripotent stem cells and their derivatives. Sci. Rep. 10, 3939. 10.1038/s41598-020-60466-9 32127560 PMC7054319

[B27] Jovanovic J. Takagi J. Choulier L. Abrescia N. G. Stuart D. I. van der Merwe P. A. (2007). alphaVbeta6 is a novel receptor for human fibrillin-1. Comparative studies of molecular determinants underlying integrin-rgd affinity and specificity. J. Biol. Chem. 282, 6743–6751. 10.1074/jbc.M607008200 17158881

[B28] Kinori M. Wehrli S. Kassem I. S. Azar N. F. Maumenee I. H. Mets M. B. (2017). Biometry characteristics in adults and children with marfan syndrome: from the marfan eye consortium of chicago. Am. J. Ophthalmol. 177, 144–149. 10.1016/j.ajo.2017.02.022 28257833 PMC5648325

[B29] Klein S. Dvornik J. L. Yarrabothula A. R. Schaniel C. (2017). A Marfan syndrome human induced pluripotent stem cell line with a heterozygous FBN1 c.4082G > A mutation, ISMMSi002-B, for disease modeling. Stem Cell Res. 23, 73–76. 10.1016/j.scr.2017.06.016 28925368

[B30] Landrum M. J. Lee J. M. Riley G. R. Jang W. Rubinstein W. S. Church D. M. (2014). ClinVar: public archive of relationships among sequence variation and human phenotype. Nucleic Acids Res. 42, D980–D985. 10.1093/nar/gkt1113 24234437 PMC3965032

[B31] Lazea C. Bucerzan S. Crisan M. Al-Khzouz C. Miclea D. Şufană C. (2021). Cardiovascular manifestations in Marfan syndrome. Med. Pharm. Rep. 94, S25–s27. 10.15386/mpr-2223 34527904 PMC8411824

[B32] Li T. Ma B. Yang H. Zhu G. Shu C. Luo M. (2021). Generation of a CRISPR/Cas9-corrected-hiPSC (NCCDFWi001-A-1) from a Marfan syndrome patient hiPSC with a heterozygous c.2613A > C variant in the fibrillin 1 (FBN1) gene. Stem Cell Res. 56, 102543. 10.1016/j.scr.2021.102543 34592602

[B33] Li X. Dong T. Li Y. Wu F. Lan F. (2019). Generation of a human iPSC line from a patient with Marfan syndrome caused by mutation in FBN1. Stem Cell Res. 36, 101414. 10.1016/j.scr.2019.101414 30870686

[B34] Li Y. Xu J. Chen M. Du B. Li Q. Xing Q. (2016). A FBN1 mutation association with different phenotypes of Marfan syndrome in a Chinese family. Clin. chimica acta; Int. J. Clin. Chem. 460, 102–106. 10.1016/j.cca.2016.06.031 27353645

[B35] Lian X. Zhang J. Azarin S. M. Zhu K. Hazeltine L. B. Bao X. (2013). Directed cardiomyocyte differentiation from human pluripotent stem cells by modulating Wnt/β-catenin signaling under fully defined conditions. Nat. Protoc. 8, 162–175. 10.1038/nprot.2012.150 23257984 PMC3612968

[B36] Lin A. Kang X. Xu Y. Feng X. Zhang S. Zhao H. (2022). Human induced pluripotent stem cells derived from a patient with a mutation of FBN1c.1858C > T (p. Pro620Ser). Stem Cell Res. 61, 102759. 10.1016/j.scr.2022.102759 35339882

[B37] Liu R. Weng G. Zheng F. Chen J. Wang K. Han J. (2024). Generation of an integration-free induced pluripotent stem cell line, FJMAi001-A, from a Marfan syndrome patient with a heterozygous mutation c.2777G > A (p.Cys926Tyr) in FBN1. Stem Cell Res. 81, 103591. 10.1016/j.scr.2024.103591 39515109

[B38] Ma B. Luo M. Yang H. Li T. Liu W. Xu F. (2020). Generation of a human induced pluripotent stem cell line (NCCDFWi001-A) from a Marfan syndrome patient carrying two FBN1 variants (c.2613A > C and c.684_736 + 4del). Stem Cell Res. 42, 101690. 10.1016/j.scr.2019.101690 31901832

[B39] Majesky M. W. (2007). Developmental basis of vascular smooth muscle diversity. Arterioscler. Thromb. Vasc. Biol. 27, 1248–1258. 10.1161/atvbaha.107.141069 17379839

[B40] Mateizel I. Spits C. De Rycke M. Liebaers I. Sermon K. (2010). Derivation, culture, and characterization of VUB hESC lines. Vitro Cell Dev. Biol. Anim. 46, 300–308. 10.1007/s11626-010-9284-4 20224973

[B41] Milewicz D. M. Braverman A. C. De Backer J. Morris S. A. Boileau C. Maumenee I. H. (2021). Marfan syndrome. Nat. Rev. Dis. Prim. 7, 64. 10.1038/s41572-021-00298-7 34475413 PMC9261969

[B42] Nakamura K. Dalal A. R. Yokoyama N. Pedroza A. J. Kusadokoro S. Mitchel O. (2023). Lineage-specific induced pluripotent stem cell-derived smooth muscle cell modeling predicts integrin alpha-V antagonism reduces aortic root aneurysm formation in marfan syndrome mice. Arterioscler. Thromb. Vasc. Biol. 43, 1134–1153. 10.1161/atvbaha.122.318448 37078287 PMC10330156

[B43] Närvä E. Autio R. Rahkonen N. Kong L. Harrison N. Kitsberg D. (2010). High-resolution DNA analysis of human embryonic stem cell lines reveals culture-induced copy number changes and loss of heterozygosity. Nat. Biotechnol. 28, 371–377. 10.1038/nbt.1615 20351689

[B44] Pan Z. Wang H. Wang H. Liu Y. Liang P. (2021). Generation of an induced pluripotent stem cell line from a patient carrying FBN1/c.6734 G > A mutation. Stem Cell Res. 55, 102459. 10.1016/j.scr.2021.102459 34298435

[B45] Park J. W. Yan L. Stoddard C. Wang X. Yue Z. Crandall L. (2017). Recapitulating and correcting marfan syndrome in a cellular model. Int. J. Biol. Sci. 13, 588–603. 10.7150/ijbs.19517 28539832 PMC5441176

[B46] Peeters S. Fedoryshchenko I. Rabaut L. Verstraeten A. Loeys B. L. (2023). Generation of an induced pluripotent stem cell (iPSC) line of a Marfan syndrome patient with a pathogenic FBN1 c.5372G > A (p.Cys1791Tyr) variant. Stem Cell Res. 68, 103050. 10.1016/j.scr.2023.103050 36801568

[B47] Pyeritz R. E. (2000). The Marfan syndrome. Annu. Rev. Med. 51, 481–510. 10.1146/annurev.med.51.1.481 10774478

[B48] Qin Z. Sun L. Sun X. Gao X. Su H. (2021). Reprogramming of a human induced pluripotent stem cell line from a Marfan syndrome patient harboring a heterozygous mutation of c.2939G > A in FBN1 gene. Stem Cell Res. 51, 102163. 10.1016/j.scr.2021.102163 33450697

[B49] Quarto N. Leonard B. Li S. Marchand M. Anderson E. Behr B. (2012a). Skeletogenic phenotype of human Marfan embryonic stem cells faithfully phenocopied by patient-specific induced-pluripotent stem cells. Proc. Natl. Acad. Sci. U. S. A. 109, 215–220. 10.1073/pnas.1113442109 22178754 PMC3252902

[B50] Quarto N. Li S. Renda A. Longaker M. T. (2012b). Exogenous activation of BMP-2 signaling overcomes TGFβ-mediated inhibition of osteogenesis in Marfan embryonic stem cells and Marfan patient-specific induced pluripotent stem cells. Stem Cells 30, 2709–2719. 10.1002/stem.1250 23037987

[B51] Sakai L. Y. Keene D. R. Engvall E. (1986). Fibrillin, a new 350-kD glycoprotein, is a component of extracellular microfibrils. J. Cell Biol. 103, 2499–2509. 10.1083/jcb.103.6.2499 3536967 PMC2114568

[B52] Sengle G. Sakai L. Y. (2015). The fibrillin microfibril scaffold: a niche for growth factors and mechanosensation? Matrix Biol. 47, 3–12. 10.1016/j.matbio.2015.05.002 25957947

[B53] Sharma T. Gopal L. Shanmugam M. P. Bhende P. S. Agrawal R. Shetty N. S. (2002). Retinal detachment in Marfan syndrome: clinical characteristics and surgical outcome. Retina 22, 423–428. 10.1097/00006982-200208000-00005 12172108

[B54] Spitalieri P. Marini M. Scioli M. G. Murdocca M. Longo G. Orlandi A. (2021). Effects of simulated microgravity on wild type and marfan hiPSCs-derived embryoid bodies. Cell. Mol. Bioeng. 14, 613–626. 10.1007/s12195-021-00680-1 34900014 PMC8630351

[B55] Stenson P. D. Ball E. V. Mort M. Phillips A. D. Shiel J. A. Thomas N. S. (2003). Human gene mutation database (HGMD): 2003 update. Hum. Mutat. 21, 577–581. 10.1002/humu.10212 12754702

[B56] Stheneur C. Faivre L. Collod-Béroud G. Gautier E. Binquet C. Bonithon-Kopp C. (2011). Prognosis factors in probands with an FBN1 mutation diagnosed before the age of 1 year. Pediatr. Res. 69, 265–270. 10.1203/PDR.0b013e3182097219 21135753

[B57] Takahashi K. Tanabe K. Ohnuki M. Narita M. Ichisaka T. Tomoda K. (2007). Induction of pluripotent stem cells from adult human fibroblasts by defined factors. Cell 131, 861–872. 10.1016/j.cell.2007.11.019 18035408

[B58] Takahashi K. Yamanaka S. (2006). Induction of pluripotent stem cells from mouse embryonic and adult fibroblast cultures by defined factors. Cell 126, 663–676. 10.1016/j.cell.2006.07.024 16904174

[B59] Takeda N. Inuzuka R. Maemura S. Morita H. Nawata K. Fujita D. (2018). Impact of pathogenic FBN1 variant types on the progression of aortic disease in patients with marfan syndrome. Circ. Genom Precis. Med. 11, e002058. 10.1161/circgen.117.002058 29848614

[B60] Thomson J. A. Itskovitz-Eldor J. Shapiro S. S. Waknitz M. A. Swiergiel J. J. Marshall V. S. (1998). Embryonic stem cell lines derived from human blastocysts. Science 282, 1145–1147. 10.1126/science.282.5391.1145 9804556

[B61] Vacante F. Venkateshappa R. Htet M. Yan C. Wu J. C. (2024). Generation of Marfan syndrome-specific induced pluripotent stem cells harboring FBN1 mutations. Stem Cell Res. 80, 103518. 10.1016/j.scr.2024.103518 39096853

[B62] Van Den Heuvel L. J. F. Peeters S. Meester J. A. N. Perik M. Coucke P. Loeys B. L. (2023). A generated induced pluripotent stem cell (iPSC) line (CMGANTi005-A) of a Marfan syndrome patient with an FBN1 c.7754T > C (p.Ile2585Thr) variation. Stem Cell Res. 67, 103032. 10.1016/j.scr.2023.103032 36708686

[B63] Verlinsky Y. Strelchenko N. Kukharenko V. Rechitsky S. Verlinsky O. Galat V. (2005). Human embryonic stem cell lines with genetic disorders. Reprod. Biomed. Online 10, 105–110. 10.1016/s1472-6483(10)60810-3 15705304

[B64] Wu S. Zhang Z. Wang L. Yu J. (2021). Generation of a human iPSC line QDMHi001-A from a patient with Marfan syndrome carrying a heterozygous c.6772 T > C variant in FBN1. Stem Cell Res. 54, 102390. 10.1016/j.scr.2021.102390 34087733

[B65] Yang Q. Zhou X. Zhou H. Li L. Du J. Lu G. (2015). Human embryonic stem cells derived from abnormal blastocyst donated by Marfan syndrome patient. Stem Cell Res. 15, 640–642. 10.1016/j.scr.2015.10.012 26987927

[B66] Yin X. Hao J. Yao Y. (2021). CRISPR/Cas9 in zebrafish: an attractive model for FBN1 genetic defects in humans. Mol. Genet. Genomic Med. 9, e1775. 10.1002/mgg3.1775 34324266 PMC8580104

[B67] Yin Xiaoke X. Wanga S. Fellows A. L. Barallobre-Barreiro J. Lu R. Davaapil H. (2019). Glycoproteomic analysis of the aortic extracellular matrix in marfan patients. Arterioscler. Thromb. Vasc. Biol. 39, 1859–1873. 10.1161/ATVBAHA.118.312175 31315432 PMC6727943

[B68] Yu Y. Shen H. Zhu J. Cao X. Li Q. Shao L. (2022). Generation of Marfan patient specific iPSCs (ICSSUi001-A) carrying a novel heterozygous mutation in FBN1 gene. Stem Cell Res. 60, 102720. 10.1016/j.scr.2022.102720 35231796

